# The Role of the NMDA Receptor in the Treatment of Psychiatric and Neurological Diseases

**DOI:** 10.3390/brainsci16080786

**Published:** 2026-07-26

**Authors:** Stan Heath, Madeline Ruzicka, Taylor McLoon, Daya Gupta, Onyinyechi Emeri, Zikora Nnabugwu, Adegoke Adeniji, Christopher Brackett, Steven Slack, Ayodeji Agbowuro

**Affiliations:** South University School of Pharmacy, South University, 709 Mall Blvd., Savannah, GA 31406, USA; stan.heath@stu.southuniversity.edu (S.H.); taylor.butler@stu.southuniversity.edu (T.M.); dgupta@southuniversity.edu (D.G.); onyinyechi.emeri@stu.southuniversity.edu (O.E.); zikoran@stu.southuniversity.edu (Z.N.); aadeniji@southuniversity.edu (A.A.); cbrackett@southuniversity.edu (C.B.); slacklab1@gmail.com (S.S.)

**Keywords:** NMDAR, neurological disorders, psychiatric disorders, therapeutic potential

## Abstract

The N-methyl-D-aspartate receptor (NMDAR) is an ionotropic glutamate receptor widely expressed in the CNS and in peripheral tissues where it mediates crucial physiological functions and its dysregulation has been linked to a host of neurological and psychiatric disorders including Alzheimer’s disease, schizophrenia, epilepsy, and chronic pain. This work highlights the therapeutic potential in targeting the NMDAR in these disease states by providing a holistic analysis of the existing literature focused on the molecular role of the receptor in applicable disease conditions. While several drugs targeting the NMDAR have been approved for clinical use, many more are in clinical and preclinical development. A significant part of this review assesses the chemical and pharmacological attributes of these molecules and provides a prognostic perspective for their use.

## 1. Introduction

The N-methyl-D-aspartate receptor (NMDAR) is an ionotropic glutamate receptor widely expressed in the CNS and in peripheral tissues where it mediates crucial physiological functions. NMDAR dysregulation has been linked to a range of psychiatric and neurological disorders, thus making the receptor an attractive drug target [1]. Several well-known compounds target the NMDAR, such as ketamine, memantine, amantadine, and dextromethorphan [2]. These drugs mostly act as antagonists to block detrimental neuronal excitation mediated by the receptor. The limited ability of the drugs to discriminate between synaptic and extrasynaptic NMDARs results in clinical adverse effects, which over time has necessitated further investigations into the activity of the receptor.

A large body of evidence shows that the NMDAR is implicated in the pathogenesis and development of a host of conditions such as Alzheimer’s disease, schizophrenia, epilepsy, and chronic pain [3,4,5,6]. This review aims to highlight the therapeutic potential in targeting the NMDAR in select disease states by providing a holistic analysis of the literature focused on the molecular role of the receptor in the disease conditions.

## 2. An Overview of the NMDA Receptor

### 2.1. Structure

**Figure 1:**
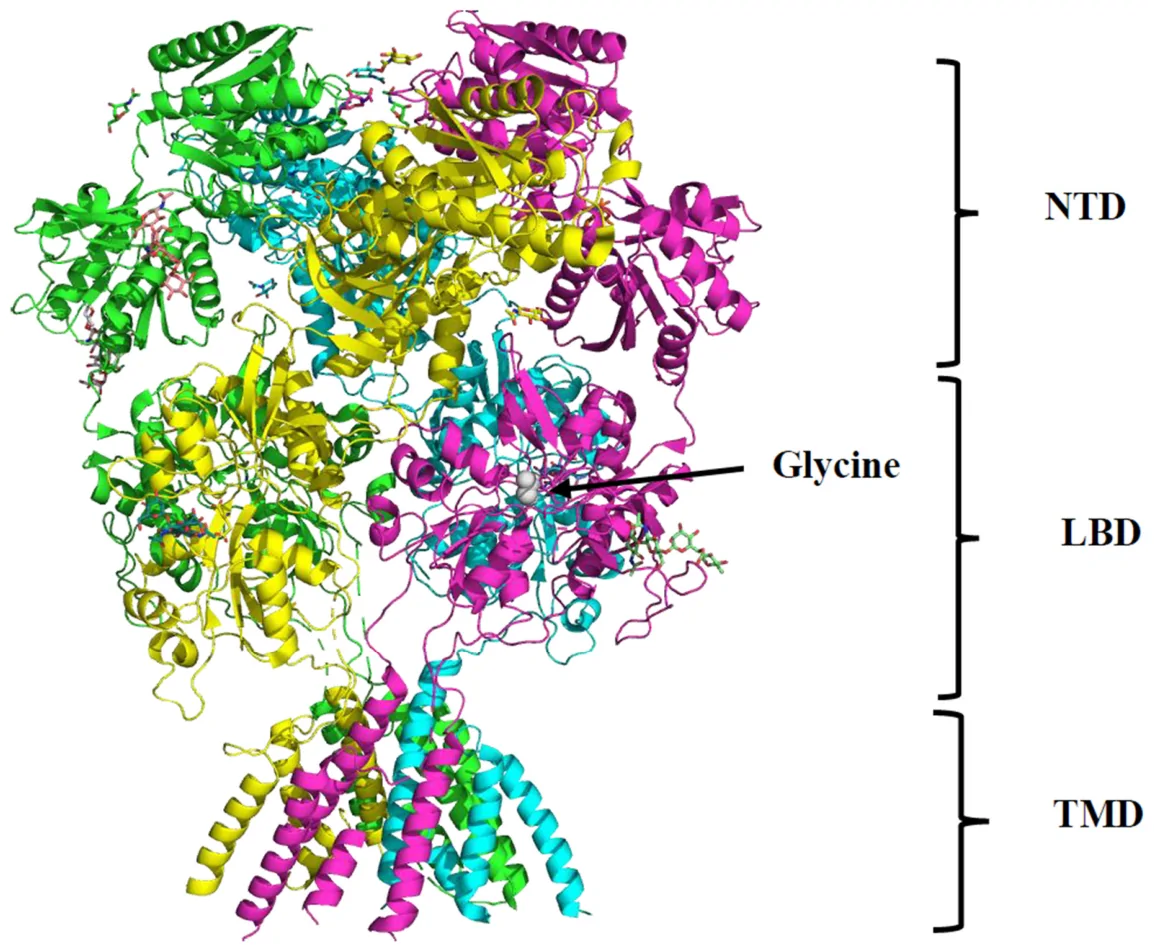
A cartoon structure of GluN1a/GluN2B NMDAR showing the amino terminal domain (NTD), ligand-binding domain (LBD), and transmembrane domain (TMD). A molecule of glycine (gray spheres) is shown bound to the ligand-binding domain. PDB ID: 4PE5 [26].

The NMDAR is a heterotetramer consisting of a combination of GluN1, GluN2A-D, and GluN3A-B subunits. Each receptor has two GluN1 subunits, which can be expressed as eight different splice variants, and two different GluN2 or GluN3 subunits [7]. The GluN2 subunit expression exhibits both spatial and temporal patterns, as GluN2B is more commonly observed in early developmental stages and contributes to synaptic plasticity and connectivity. Conversely, GluN2A expression increases later in the developmental stages and is associated with more stable neural connections [8]. Independent of the type, each subunit shares the same general structural features: the extracellular amino terminal domain (NTD), the extracellular ligand-binding domain (LBD), the transmembrane domain (TMD), and the C-terminal intracellular domain (CTD) (Figure 1) [9].

The GluN2 subunit is responsible for binding to glutamate or N-methyl-D-aspartate, whereas GluN1 binds to the co-agonist, glycine or D-serine. Concomitant binding of the primary agonist and the co-agonist is necessary for NMDAR activation. It has been observed that D-serine acts as the co-agonist for synaptic receptors, whereas glycine is used in extrasynaptic receptors [10]. Interestingly, NMDARs composed of GluN1/GluN3 subunits do not require glutamate for activation, and are not considered glutamate receptors [11]. The ligand-binding domain across the different subunits share a similar clamshell-like conformation, composed of two polypeptide sequences, S1 and S2, with each polypeptide comprising one half of the clamshell [12]. The specific ligand will bind in the cleft between these two parts, primarily interacting with residues found in polypeptide segment S1. Upon ligand binding, the receptor adopts a closed conformation that acts as a trigger to initiate ion channel opening [13].

The transmembrane domain is divided into four segments, denoted M1-4. M1, M3, and M4 are membrane-spanning helices, and the M2 loop serves as the gate. A key feature of the M2 loop is the conserved Q/R/N site that resides at the apex of the loop, and is critical for both Ca^2+^ permeability and Mg^2+^ blockage [14]. The transmembrane helix, M3, has been shown to be on the inside of the channel, and consequently is the most critical for ion transport. The conformational change that occurs in the ligand-binding domain upon glutamate and glycine binding causes a rotation in the M3 helices away from the center of the channel [15,16]. The open channel is primarily responsible for allowing Ca^2+^ influx, but is also permeable to Na^+^ and K^+^ influx and efflux, respectively. Ion flux through the NMDAR only occurs after Mg^2+^ has been removed from the channel, secondary to an α-amino-3-hydroxy-5-methyl-4isoxazolepropionic acid (AMPA) receptor-mediated membrane depolarization [17].

The intracellular C-terminal domain (CTD) is highly variable amongst the different subunits of NMDAR, but is involved in trafficking and localization of the receptor [18,19]. Several proteins are known to interact with the CTD of GluN1, an example is postsynaptic density protein-95 (PSD-95), which regulates NMDAR channel gating and surface expression. Other prominent ones include calmodulin, which mediates Ca^2+^-dependent inactivation of the NMDAR and contributes to receptor trafficking and synaptic plasticity, α-Actinin-2, which regulates Ca^2+^-dependent channel activity and receptor localization, and the neurofilament subunit, NF-L [14,20,21,22]. The CTD is also a target for kinases and phosphatases, which have been observed to control cellular distribution of NMDAR as well as long-term potentiation [23,24,25].

### 2.2. Physiological Functions

**Figure 2:**
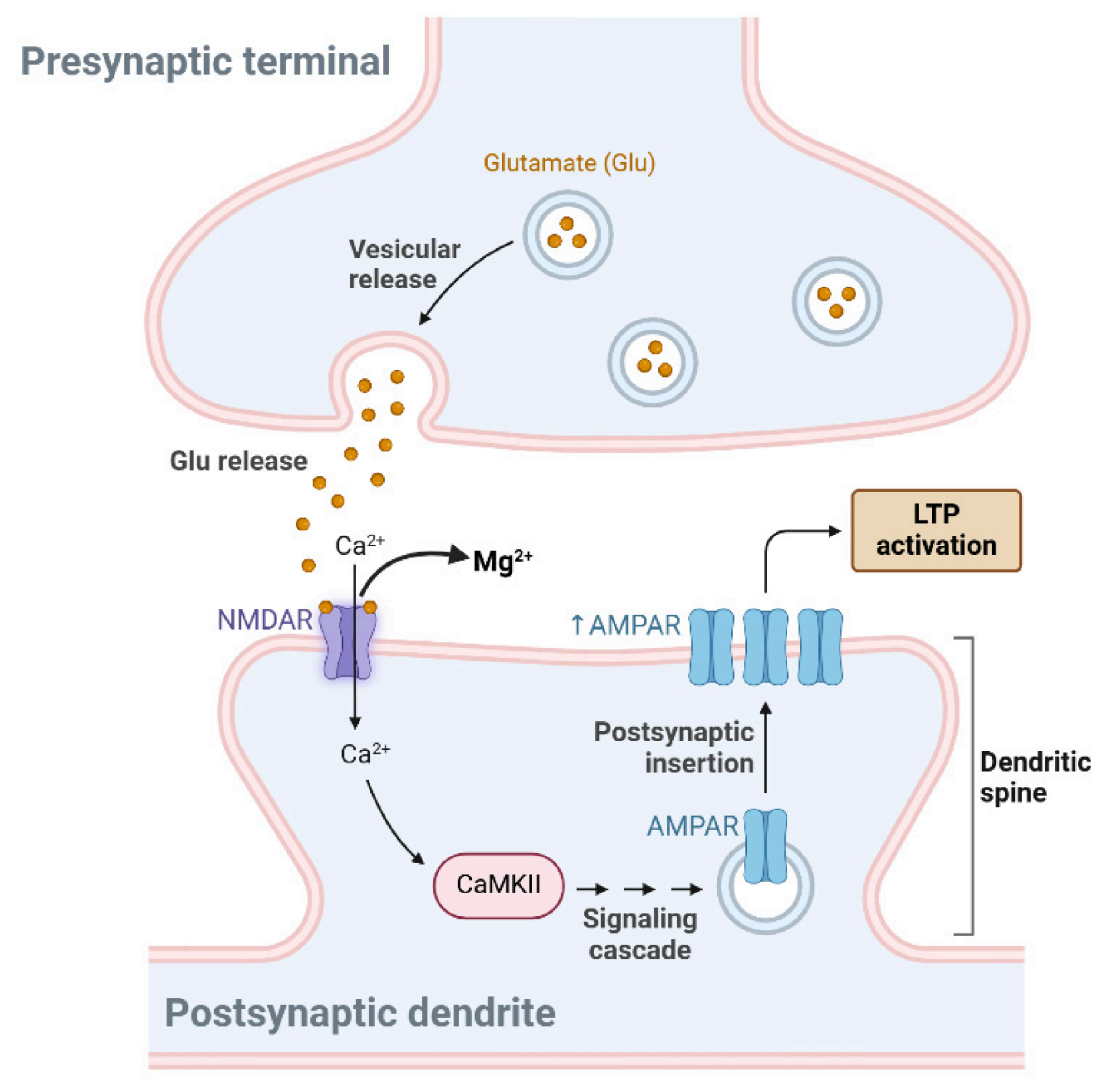
Activation of NMDAR-dependent long-term potentiation (LTP). Presynaptically released glutamate binds postsynaptic NMDAR, leading to depolarization that results in calcium influx and the subsequent activation of various complex signaling pathways catalyzed by calcium/calmodulin-dependent protein-kinase II (CaMKII). This causes an increase in the number of α-amino-3-hydroxy-5-methyl-4-isoxazole propionic acid receptors (AMPARs) in the postsynaptic plasma membrane, facilitating LTP activation. Figure was created in https://BioRender.com.

Upon biosynthesis in the endoplasmic reticulum, the heterotetrametric NMDAR is transported to the cell surface and subsequently trafficked to synapses and extrasynaptic regions of neurons [27]. The activation of NMDAR is initiated by presynaptic release of glutamate binding at the ligand-binding domains (LBDs) and the NTD. This causes postsynaptic neuron depolarization which results in a voltage-dependent reversal of the magnesium (Mg^2+^)-induced blockade of the ion channels leading to calcium ion influx in postsynaptic dendritic spines [28,29]. Under homeostatic conditions, the resultant effect is an activation of various cellular signaling pathways that manifest as the physiological functions of the NMDAR (Figure 2) [30,31]. The primary physiological function of the NMDAR is mediating synaptic plasticity and memory function. This is achieved by an influx of calcium ions that happens only when both presynaptic and postsynaptic activity occur concurrently, thus facilitating long-term potentiation (LTP) and long-term depression (LTD) in the brain. LTP processes in particular are seen as a plasticity change that is fundamental to learning and long-term memory functions. Other physiological roles of the NMDAR include the regulation of neuronal migration, synaptogenesis, and synaptic maturation [1]. Malfunctioning of the NMDAR causes neuronal excitotoxicity that is implicated in many neurological and psychiatric disorders, hence the therapeutic usage of antagonists of the receptor, like ketamine.

## 3. Targeting the NMDAR in Disease Conditions

**Figure 3:**
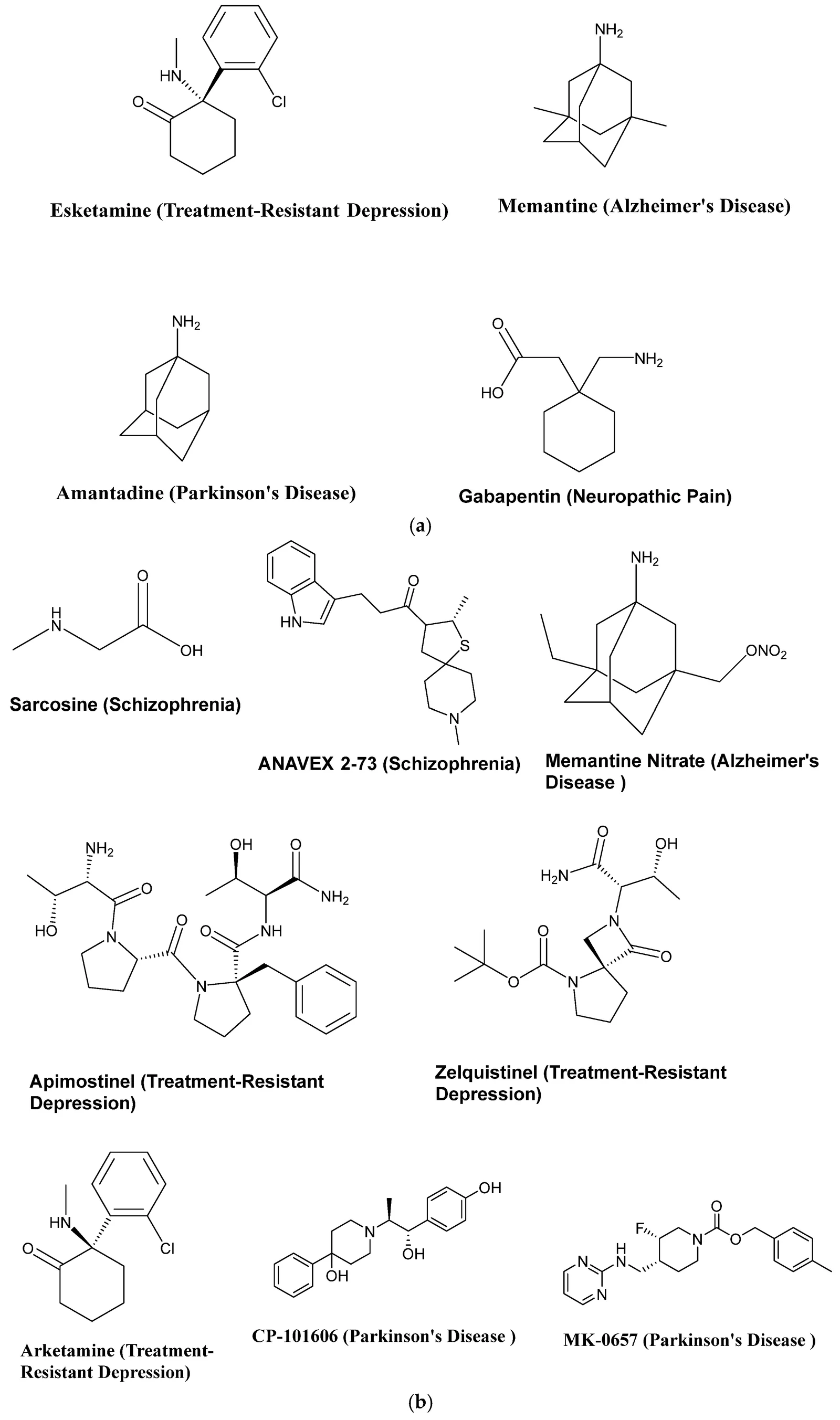
(**a**) Chemical structures of select clinically used NMDAR modulators and their indications. (**b**) Chemical structures of select investigational NMDAR modulators and their intended indications.

This section discusses select disease states with underlying NMDAR malfunction and the various NMDAR-targeted molecules used in their treatment. The chemical structures of the molecules discussed are presented as Figure 3a,b. Additionally, a summary of the clinical development status of the molecules and their therapeutic modalities is presented in Table 1. Across several disease states, ketamine and esketamine are presented as potential treatment modalities. It should be noted that these drugs are schedule III-controlled substances in the United States and carry the potential for abuse. They are therefore subject to laws and regulations governing their distribution and administration [32,33].

### 3.1. Schizophrenia

Schizophrenia is characterized by a combination of positive symptoms, such as delusions, hallucinations, bizarre behaviors, and formal thought disorder, as well as negative symptoms, including social withdrawal and anhedonia. Cognitive impairments, which encompass deficits in attention, working memory, and executive function, are also common. Based on the observation that NMDAR antagonists like ketamine and phencyclidine produce schizophrenia-like symptoms, schizophrenia has been strongly linked to NMDAR hypofunction [34]. NMDAR hypofunction results in glutamate dysregulation, which disrupts the excitatory–inhibitory balance in the brain. This leads to decreased GABAergic transmission, thereby reducing inhibitory control and causing excessive excitatory activity in certain brain regions [35]. Additionally, NMDAR dysfunction impacts dopamine neuron activity in the nigrostriatal, mesolimbic, and mesocortical pathways, contributing to dopamine dysregulation [36]. It is important to highlight that each NMDAR subunit exhibits distinct pathophysiological functions when interacting with antagonists like ketamine. Khlestova et al. utilized data that characterize ketamine’s ability to block different NMDAR subtypes to deduce the subunit that is mostly involved in schizophrenia. Based on their analysis, at psychotogenic concentrations in humans, ketamine preferentially blocks GluN2C subunit-containing receptors with less effects on GluN2A-, GluN2B-, and GluN2D-containing receptors [34]. This information becomes relevant in the pursuit of NMDAR-active molecules with anti-schizophrenic potential.

Glutamatergic drugs, including glycine modulators and NMDAR enhancers, are currently being explored as potential treatments for schizophrenia. A significant focus is on Glycine Transporter-1 (GlyT1) inhibitors, which increase synaptic glycine levels, enhancing NMDAR activity. An example of this drug class is sarcosine, also known as N-methylglycine. As an investigative adjunctive therapy for schizophrenia, sarcosine produced significant improvements in positive symptoms, negative symptoms, and speed of processing in 22 stabilized patients [37]. Another drug class of interest are the D-amino acid oxidase (DAAO) inhibitors. D-serine acts as a co-agonist at the glycine site, directly activating NMDARs. DAAO catalyzes the breakdown of D-serine to hydroxypyruvate and is highly expressed in the cerebellum and brainstem. Sodium Benzoate, a DAAO inhibitor, boosts endogenous D-serine levels by inhibiting its breakdown [38]. Notably, in a Phase 2 clinical trial, luvadaxistat, a DAAO inhibitor was shown to have positive effects on cognition in patients with schizophrenia even though it did not produce a significant improvement in the negative symptoms of schizophrenia [39]. Given that D-serine is poorly absorbed, administering it in conjunction with DAAO inhibitors may also increase its bioavailability and potentially enhance NMDAR function and improve the symptoms of schizophrenia.

Lastly, ANAVEX3-71, a sigma-1 receptor agonist, is currently being investigated for treating schizophrenia [40]. The sigma-1 receptor is known to modulate NMDAR activity [41]. Upon activation, the sigma-1 receptor translocates to the plasma membrane where it physically interacts with NMDAR GluN2 subunits. This interaction enhances receptor channel activity and potentiates calcium influx. Sigma-1 receptor agonists amplify NMDAR-mediated currents in hippocampal neurons, facilitate long-term potentiation, and promote precognitive effects [42,43].

### 3.2. Treatment-Resistant Depression (TRD)

Depression is a psychiatric disorder that affects an individual’s mood, behavior, and overall health. Depression embodies mood shifts toward persistent feeling of sadness, inability to experience pleasure, and in many cases, suicidal ideation. According to the World Health Organization (WHO), more than 720,000 people die by suicide every year [44]. This in part is attributable to the delayed response rate and treatment failure associated with conventional antidepressants. Treatment-Resistant Depression is defined as failure to respond to two or more first-line antidepressant regimens despite adequate dose and duration and adherence to treatment [45], these first-line agents being selective serotonin reuptake inhibitors (SSRIs) or serotonin and norepinephrine reuptake inhibitors (SNRIs), which specifically act on the monoaminergic system.

Targeting glutamatergic neurotransmission via the NMDAR has been shown to produce therapeutically beneficial antidepressant effects in TRD. Mechanistically, the antidepressant effects of NMDAR antagonists like (R,S)-ketamine are mediated through the preferential suppression of NMDAR GluN2A subunit and subsequent activation of the AMPAR [46,47]. In 2019, esketamine, the (S)-enantiomer of ketamine was approved by the U.S. Food and Drug Administration (FDA) for TRD, to be used in combination with an oral antidepressant. Its use was subsequently expanded to monotherapy in January 2025, allowing esketamine to be used as a standalone therapy for refractory depression [48]. However, of the two enantiomers of ketamine, only esketamine is currently used for TRD, but there is growing evidence that the R- enantiomer (arketamine) produces greater potency and longer-lasting antidepressant effects compared to esketamine and may be more clinically beneficial [46,48].

Apimostinel and Zelquistinel are orally available NMDAR modulators being developed for the treatment of major depressive disorder. Apimostinel is a modified tetrapeptide with enhanced metabolic stability. Clinical and preclinical investigations show that it exhibits potent and sustained antidepressant effects without the psychotomimetic side effects associated with ketamine [49]. Zelquistinel on the other hand is non-peptidic, but equally produces desirable bioactivity [50]. Given the robust antidepressant effects and relative clinical safety of these molecules, they are promising candidates for TRD.

### 3.3. Alzheimer’s Disease (AD)

Alzheimer’s disease is a neurodegenerative disease that gradually diminishes memory and cognitive abilities over time as neurotoxic substances such as amyloid-beta and hyperphosphorylated tau proteins accumulate in the brain and disrupt nerve cell connections [51]. The NMDAR typically helps with learning and memory inside healthy synapses; however, in AD, amyloid-beta oligomers trigger NMDAR hyperactivity causing excessive Ca^2+^ entry into the neurons, which eventually leads to neuronal death. As a result, synaptic NMDARs that support neuronal survival consequently become underactive. This imbalance drives the brain atrophy and cognitive decline that characterize AD [52].

Memantine is an NMDAR antagonist approved for the treatment of moderate to severe AD. It blocks the NMDAR in a use-dependent manner primarily impacting hyperactivated receptors [53,54,55]. As monotherapy, memantine has proven to be beneficial over short and long durations, and the addition of an acetylcholinesterase inhibitor provides additive clinical benefits without negatively impacting side effects. However, it has limited benefits for individuals categorized in the mild stage of this disease [56]. Current research efforts are geared at developing NMDAR antagonists with improved efficacy and coverage compared to memantine. MN-08 (memantine nitrate) is an analog of memantine found to be more potent than memantine in cognition enhancement and increasing cerebral blood flow. It functions via a dual mechanism involving NMDAR antagonism and dilatation of cerebral blood vessels by nitric oxide (NO) release [57,58]. Similarly, RL-208, an orally bioavailable NMDA receptor antagonist, significantly improved cognitive performance in mouse models of AD [59]. These agents are in various stages of drug development and represent potentially viable candidates for the treatments for AD.

### 3.4. Parkinson’s Disease (PD)

Parkinson’s disease is a progressive movement disorder characterized by tremors, rigidity, bradykinesia, and impaired balance. These symptoms worsen over time and may eventually result in death. The incidence and prevalence of PD has increased dramatically over the past decade despite the availability of efficacious therapies [60]. It is primarily linked to a substantial loss of dopaminergic neurons in the nigrostriatal pathway of the brain, which is largely caused by the deposition of aggregated α-synuclein (Lewy bodies) [61]. Additionally, overactivity of glutamatergic NMDAR has been implicated in the pathogenesis of PD. As a result, NMDAR modulators have been leveraged for the treatment of the disease [62]. Amantadine, an NMDAR antagonist was serendipitously discovered to be safe and efficacious in improving the symptoms of PD. However, it is often used as an adjunctive therapy for levodopa-induced dyskinesias and its efficacy is usually for a short duration, lasting weeks to months [63]. With the aim of improving treatment outcomes, several NMDAR modulators are being investigated for PD treatment. Examples include selective NMDAR antagonists such as CP-101606 and MK-0657 with proven benefits in preclinical models of PD, albeit their clinical translation has been challenging [64]. However, with further optimization efforts, these molecules have the potential to produce clinically relevant drugs.

### 3.5. Neuropathic Pain

Neuropathic pain is chronic pain caused by malfunctioning of the somatosensory system and affects approximately 7–10% of the general population. In contrast to nociceptive pain, which is an adaptive process that alerts an individual to potential tissue damage, neuropathic pain affects the pain-signaling system itself [65]. Central to this mechanism is the recruitment and overactivation of presynaptic NMDAR to enhance glutamate release and drive an increase in neuronal excitability [66]. This increases pain signaling such that even moderate stimulation becomes excruciating. As a result, NMDAR modulators like ketamine and gabapentin are potent pain medications [67]. Gabapentin and pregabalin are clinically used pain medications that act by disrupting the interaction of α2δ-1 subunit of calcium channels with the NMDAR, which blocks nerve injury-induced potentiation of NMDAR activity [66].

The newer strategies currently under investigation include GluN2B-selective antagonists that selectively interfere in pain pathways while sparing normal brain functions. This is expected to produce more effective pain relief with a potentially better side effect profile compared to nonselective antagonists. A notable example is the previously discussed CP-101606, a GluN2B-selective antagonist which has also been under investigation for treatment of chronic pain. As hypothesized, CP-101606 produced relief of chronic pain outlasting the treatment period with no side effects in a rat model of neuropathic pain [68]. This shift to subunit-specific targeting is a more precise form of pain management and may offer superior solutions to the millions of people suffering from neuropathic pain globally.

### 3.6. Epilepsy

Epilepsy is a neurological disorder characterized by spontaneous seizures that have multiple neurologic, cognitive, and psychosocial consequences. It is specifically defined as two or more unprovoked seizures occurring more than 24 h apart; a single unprovoked seizure if recurrence risk is high (>60% over the next 10 years); or a diagnosis of an epileptic syndrome [69]. Epilepsy affects about 1% of the U.S. population each year and up to 65 million people worldwide [70]. On a molecular level, neuronal hyperexcitability resulting from NMDAR malfunction is widely known to be a major component of the pathogenesis of epilepsy [71]. Thus, NMDAR antagonists play beneficial roles in epilepsy treatment. For instance, ketamine produces a decrease in seizure burden in patients with super-refractory status epilepticus [72].

While Gamma-aminobutyric acid (GABA) is the main inhibitory neurotransmitter in the brain and the foremost target for second generation antiepileptics, the need to accurately time the administration of GABA-targeted drugs limits their overall efficacy. This is particularly important during a status epilepticus episode. In the first 30 min of continuous epileptic seizure activity, GABA-A receptors undergo internalization, resulting in fewer available receptors at the cell surface [73]. At this point, drugs that block NMDA receptors and glutamate-mediated excitation (such as ketamine) become necessary, as GABA enhancing drugs such as benzodiazepines become less effective. Also, magnesium sulfate is used to treat status epilepticus, particularly refractory status epilepticus, by acting as an NMDA receptor antagonist. While promising, especially in specific conditions like febrile illness-related epilepsy syndrome, more research is needed to fully establish the efficacy of magnesium sulfate therapy across all types of status epilepticus [74].

## 4. Conclusions

The NMDAR plays an important role in regulating crucial neuronal functions. It regulates neuronal Ca^2+^ influx through the binding of glutamate and the co-agonists, glycine and d-serine. Aberrant NMDAR signaling through hyper/hypo-functioning or dysregulation of its interactions with other neurotransmitter-responsive biomolecules has been linked to several neuropsychiatric disorders such as schizophrenia, depression, Alzheimer’s disease, Parkinson’s disease, neuropathic pain, and epilepsy. These diseases impact millions of people globally and remain an area with a significant need for more efficacious and well tolerated therapeutic agents. The involvement of the NMDAR signaling pathway in a wide variety of neurologic disease states and the potential therapeutic versatility of NMDAR targeting agents has led to a surge in interest in identifying promising lead compounds.

Advances in techniques used for biomolecular structure elucidation have enabled a more comprehensive understanding of the NMDAR structure–function relationship, which is currently driving the development of NMDAR-targeted molecules. These molecules include NMDAR modulators, subunit-selective compounds, and co-agonists, some of which are in varying phases of drug development. To fully optimize the use of these compounds, ongoing efforts should be redoubled to further elucidate the precise role of NMDAR in these disorders, the involvement of varying receptor subunit, its position in the pathological cascade, and any potential redundancies associated with its function in the disease state.

Based on data emanating from preclinical and clinical investigations, we envisage that more potent and safer NMDAR-targeted molecules will be available in the clinic in the near future.

## Figures and Tables

**Table 1:** Current clinical development status of NMDA receptor-targeting drug candidates and therapeutic modalities across select neurological and psychiatric disorders.

Disorder	Drug Candidate	Molecular Target/Mechanism	Development Stage	Comments
**Schizophrenia**	Sarcosine (N-methylglycine)	GlyT1 inhibitor; increases synaptic glycine and enhances NMDAR function	Clinical (Phase II/adjunct studies)	Demonstrated improvement in positive, negative and cognitive symptoms as adjunctive therapy.
	Sodium Benzoate	D-amino acid oxidase (DAAO) inhibitor; increases endogenous D-serine	Clinical (Phase II)	Multiple clinical studies show benefit as adjunctive treatment.
	Luvadaxistat	Potent DAAO inhibitor	Phase II completed	Improved cognition but failed to significantly improve negative symptoms.
	ANAVEX3-71	Sigma-1 receptor agonist and M1 muscarinic receptor agonist; indirectly modulates NMDAR	Phase II	Under clinical development for schizophrenia, Alzheimer’s disease and frontotemporal dementia.
**Treatment-Resistant Depression**	Esketamine	Noncompetitive NMDAR antagonist	FDA approved	Approved for TRD (2019); monotherapy approval expanded in 2025.
	Ketamine (racemate)	Noncompetitive NMDAR antagonist	Approved (selected indications)/clinical use	Widely used off-label for TRD and approved in several countries for depression-related indications.
	Arketamine (R-ketamine)	Noncompetitive NMDAR antagonist	Phase III/late clinical development	May provide longer-lasting antidepressant effects than esketamine.
	Apimostinel	NMDAR positive allosteric modulator (rapastinel analogue)	Phase II	Rapid antidepressant effects with minimal psychotomimetic adverse effects.
	Zelquistinel	Oral NMDAR positive allosteric modulator	Phase II	Oral neuroplastogen with sustained antidepressant activity.
**Alzheimer’s disease**	Memantine	Low-affinity uncompetitive NMDAR antagonist	FDA approved	Standard therapy for moderate-to-severe AD.
	Memantine nitrate	Dual NMDAR antagonist and nitric oxide donor	Phase II	Improved cognition and cerebral blood flow in preclinical and early clinical studies.
	RL-208	Orally active NMDAR antagonist	Preclinical	Improved cognition in AD mouse models.
**Parkinson’s disease**	Amantadine	Weak NMDAR antagonist	FDA Approved	Used for Parkinson’s disease and levodopa-induced dyskinesia.
	CP-101606	Selective GluN2B antagonist	Phase II	Demonstrated efficacy preclinically; development limited by adverse effects.
	MK-0657	Selective GluN2B antagonist	Phase I/II	Did not demonstrate clinically meaningful motor improvement.
**Neuropathic pain**	Ketamine	Noncompetitive NMDAR antagonist	Clinical use	Effective for refractory neuropathic pain and perioperative pain management.
	Gabapentin	Indirect NMDAR modulation via α2δ-1 calcium channel interaction	FDA approved	Standard first-line therapy for neuropathic pain.
	Pregabalin	Indirect NMDAR modulation via α2δ-1 calcium channel interaction	FDA approved	Standard first-line therapy for neuropathic pain.
	CP-101606	GluN2B-selective antagonist	Preclinical/early clinical	Produced prolonged analgesia in experimental models.
**Epilepsy**	Ketamine	NMDAR antagonist	Clinical use	Used for refractory and super-refractory status epilepticus.
	Magnesium sulfate	Physiological NMDAR channel blocker	Clinical use	Utilized particularly in refractory status epilepticus; evidence continues to evolve.

## Data Availability

No new data were created during this review.

## Abbreviations

The following abbreviations are used in this manuscript:

AD	Alzheimer’s Disease
AMPA	α-amino-3-hydroxy-5-methyl-4isoxazolepropionic acid
CaMKII	Calcium/calmodulin-dependent protein-kinase II
CTD	C-terminal domain
DAAO	D-amino acid oxidase
FDA	Food and Drug Administration
GABA	Gamma-aminobutyric acid
GlyT1	Glycine Transporter-1
LBD	Ligand-binding domain
LTD	Long-term depression
LTP	Long-term potentiation
NMDAR	N-methyl-D-aspartate receptor
NTD	Amino terminal domain
PD	Parkinson’s Disease
SNRIs	Serotonin and norepinephrine reuptake inhibitors
SSRIs	Selective serotonin reuptake inhibitors
TMD	Transmembrane domain
TRD	Treatment-Resistant Depression
WHO	World Health Organization
